# Identification of miRNA–mRNA–TFs Regulatory Network and Crucial Pathways Involved in Tetralogy of Fallot

**DOI:** 10.3389/fgene.2020.00552

**Published:** 2020-06-12

**Authors:** Guoling You, Bailing Zu, Bo Wang, Qihua Fu, Fen Li

**Affiliations:** ^1^Department of Laboratory Medicine, Shanghai Children’s Medical Center, Shanghai Jiao Tong University School of Medicine, Shanghai, China; ^2^Department of Cardiology, Shanghai Children’s Medical Center, Shanghai Jiao Tong University School of Medicine, Shanghai, China

**Keywords:** congenital heart disease, differential expression genes, microRNAs, regulatory networks, Tetralogy of Fallot

## Abstract

Tetralogy of Fallot (TOF) is the most common cyanotic congenital heart disease. However, its pathogenesis remains unknown. To explore key regulatory connections and crucial pathways underlying the TOF, gene or microRNA expression profile datasets of human TOF were obtained from the National Center for Biotechnology Information (NCBI) Gene Expression Omnibus (GEO) database. The differentially expressed mRNAs (DEmRNAs) and microRNAs (DEmiRs) between TOF and healthy groups were identified after data preprocessing, followed by Gene Ontology (GO) analysis and Kyoto Encyclopedia of Genes and Genomes (KEGG) pathway enrichment analysis. Then, we further constructed protein–protein interaction (PPI) network and subnetwork of modules. Ultimately, to investigate the regulatory network underlying TOF, a global triple network including miRNAs, mRNAs, and transcription factors (TFs) was constructed based on the integrated data. In the present study, a total of 529 DEmRNAs, including 115 downregulated and 414 upregulated DEmRNAs, and 7 significantly upregulated DemiRs, including miR-499, miR-23b, miR-222, miR-1275, miR-93, miR-155, and miR-187, were found between TOF and control groups. Furthermore, 22 hub genes ranked by top 5% genes with high connectivity and six TFs, including SRF, CNOT4, SIX6, SRRM3, NELFA, and ONECUT3, were identified and might play crucial roles in the molecular pathogenesis of TOF. Additionally, an miRNA–mRNA–TF co-regulatory network was established and indicated ubiquitin-mediated proteolysis, energy metabolism associated pathways, neurodevelopmental disorder associated pathways, and ribosomes might be involved in the pathogenesis of TOF. The current research provides a comprehensive perspective of regulatory mechanism networks underlying TOF and also identifies potential molecule targets of genetic counseling and prenatal diagnosis for TOF.

## Introduction

Cardiac development is an extremely complex process involving the integration of multiple cell lineages into the three-dimensional organ and its seamless connection to the vascular system (Liu and Olson, 2010). Proper embryological heart development and function are precisely controlled by an evolutionarily conserved network of transcription factors (TFs), which are triggered by upstream signaling systems involved in cardiac myogenesis, morphogenesis, and contractility (Olson, 2006; Liu and Olson, 2010). Abnormalities in the networks during the cardiac formation process might lead to the failure of proper cardiac differentiation, migration, and apoptosis, which finally results in congenital heart disease (CHD).

Tetralogy of Fallot (TOF) is the most common cyanotic CHD, occurring in about 3 of every 10,000 live births and accounting for 7–10% of CHD (Bailliard and Anderson, 2009). TOF is caused by the non-uniform separation between bulbos arteriosus and truncus arteriosus during the early stage of embryonic development, resulting in malformations such as obstruction of the right ventricular outflow tract, ventricular septal defect (VSD), override of the ventricular septum by the aortic root, and right ventricular hypertrophy (Bailliard and Anderson, 2009; Wang et al., 2018). The clinical, pathological, phenotypical, and biologic heterogeneity of TOF suggests the complex pathogenesis. The precise molecular pathogenesis of TOF has not been completely identified. However, the latest research is advancing the understanding of the molecular mechanisms of TOF. Today, most investigations into the pathogenesis of TOF have focused on mutations in specific protein-coding genes, such as *GATA4*, *NKX-2.5*, *JAG1*, *FOXC2*, *TBX5*, *TBX1*, etc. (Lalani and Belmont, 2014). In addition, microdeletions of chromosome 22q11.2 and several other copy number variations, including15q11.2, 1q21.1, 2p13.3, 3p25.1, and 16p13.11, are found implicated in the pathological mechanisms of TOF (Lalani and Belmont, 2014).

Recently, several studies have revealed that microRNAs (miRNAs) play crucial roles in cardiac signaling and transcriptional pathway modulating multiple cardiac development, function, and disease (Liu and Olson, 2010). Cardiac development depends on the proper spatiotemporal expression of particular miRNAs. miR-1 was the first reported miRNA to be implicated in cardiac development, which targets DLL1 and HAND2 and further promotes the differentiation of embryonic stem cells into cardiac lineage (O’Brien et al., 2012). MiR-133a-1 and miR-133a-2 double-knockout mice have abnormal expression of SRF and CCND2, leading to late embryonic and neonatal lethality due to chamber dilatation and VSD (Liu et al., 2008). Deletion of either miR-106a-363 or miR-106b-25 combined with the miR-17∼92 null allele can result in embryonic lethality, accompanied by severe VSD, atrial septal defects, and thin-walled myocardium (Ventura et al., 2008). Taken together, these studies revealed that miRNAs play vital roles in vertebrate heart development. However, a comprehensive profiling of miRNA regulatory networks in TOF has not been reported to date.

The molecular mechanism and critical regulators involved in TOF are poorly deciphered. In the present study, to investigate the regulatory networks and key pathways underlying TOF, we constructed the protein–protein interaction (PPI) and an integrative miRNA–mRNA–TF regulatory network to further elucidate the key mechanisms underlying TOF.

## Materials and Methods

### Collection of Datasets

All of the TOF datasets were obtained from the Gene Expression Omnibus (GEO) (Clough and Barrett, 2016) with the following criteria: (1) mRNA or miRNA expression profiles by microarray or RNA-seq could be available; and (2) TOF patients and healthy controls were estimated. Consequently, a total of four microarray datasets [GSE35776 (O’Brien et al., 2012), GSE26125 (Bittel et al., 2011), GSE40128 (Zhang et al., 2013), and GSE35490 (Bittel et al., 2014)] and one RNA-seq dataset [GSE36761 (Grunert et al., 2014)] were included in this study, in which GSE40128 and GSE35490 were used for its miRNA expression profile. A total of 107 samples (32 controls and 75 cases) were analyzed in this study; details are provided in Table 1.

**TABLE 1:** Microarray and RNA-seq datasets used in this study and their experimental design.

	GEO accession number	Sample size		Platform
		
		Controls	Patients	
1	GSE35776	8	16	(HuEx-1_0-st) Affymetrix Human Exon 1.0 ST Array
2	GSE36761	7	22	Illumina Genome Analyzer
3	GSE26125	5	16	CodeLink Human Whole Genome Bioarray
4	GSE40128	3	5	(miRNA-1) Affymetrix Multispecies miRNA-1 Array
5	GSE35490	8	16	(miRNA-1) Affymetrix Multispecies miRNA-1 Array

*GEO, Gene Expression Omnibus dataset.*

### Identification of Differentially Expressed mRNAs and miRNAs

For microarray data, background correction, quantile normalization, and determination of expression levels were conducted using R/Affy (Gautier et al., 2004) or Oligo (Carvalho and Irizarry, 2010) packages. Differential expression analysis of mRNAs or miRNAs between the TOF and control groups was conducted using the R/Limma package (Diboun et al., 2006).

For RNA sequencing dataset (GSE36761), adapter sequences of raw reads were trimmed by Trimmomatic (Bolger et al., 2014). STAR was utilized to align the short reads to the reference human genome (hg38) (Dobin et al., 2013). Accepted mapped reads were then summarized at gene level counts using featureCounts software (Liao et al., 2014). Differential gene expression analysis between the TOF and control groups was conducted using R package DESeq2 (Love et al., 2014).

The adjusted *P* < 0.05 and |log Fold Change (FC)| > 2 were set as the cut-off criteria for differentially expressed mRNAs (DEmRNAs), as well as differentially expressed miRNAs (DEmiRs) with thresholds of |log FC| > 1.5 and adjusted *P* < 0.05.

### miRNA–Target Regulatory Network Analysis

miRWalk software is a comprehensive database that provides predicted and validated miRNA binding sites of known genes of human, rat, cow, mouse, and dog (Dweep and Gretz, 2015). Experimentally verified miRNA–gene regulatory pairs were considered as targets of DEmiRs. Then, we extracted the genes as significant DEmRNAs by intersecting the target genes of the DEmiRs and the overlapping genes of the DEmRNAs. The miRNA–target gene regulatory network was constructed using the Cytoscape software (Shannon et al., 2003).

### Gene Ontology and Kyoto Encyclopedia of Genes and Genomes Pathway Enrichment Analysis

To further understand the gene function in TOF, Gene Ontology (GO) analysis (Ashburner et al., 2000) and Kyoto Encyclopedia of Genes and Genomes (KEGG) pathway enrichment analysis (Kanehisa and Goto, 2000) of the DEmRNAs were performed using Metascape (Zhou et al., 2019), with *P* < 0.05 as the cut-off criterion.

### PPI Network Construction and Identification of Hub Genes

The STRING (Search Tool for the Retrieval of Interacting Genes/Proteins) database provides a critical assessment and integration of PPI, including direct (physical) as well as indirect (functional) associations (Szklarczyk et al., 2015). The PPI networks of DEmRNAs were identified using the STRING database, and PPI score threshold was set as 0.4 to obtain interactions with higher confidence (>0.4). As nodes with a higher degree of connectivity contributing more to the stability of the network, the top 5% DEmRNAs ranked by the degree connectivity were defined as hub genes by using the plugin CentiScaPe (Scardoni et al., 2009). The Molecular Complex Detection (MCODE) (Bader and Hogue, 2003) plugin was applied to identify significant modules with node score cutoff = 0.2, K-Core = 4, max depth = 100 and degree cut-off = 4 set as the cut-off criteria. The PPI network was visualized by Cytoscape software (Shannon et al., 2003).

### miRNAs–mRNAs–TFs Regulatory Network Analysis

MicroRNAs can regulate gene expression at the posttranscriptional stage (Slezak-Prochazka et al., 2010), whereas TFs can activate or repress transcription at a pretranscriptional stage (Zaret and Carroll, 2011). The miRNA–target regulatory network was visualized to depict interactions between miRNAs and their potential targets using Cytoscape 3.7.1.

The plugin iRegulon (Janky et al., 2014) in Cytoscape software, which included multiple human databases of the TF–target pairs such as Transfac and Encode, was used to identify TFs regulating DEmRNAs in the miRNA–target regulatory network. In this study, the parameters for TF motif enrichment analysis were set as the minimum identity between orthologous genes ≥0.05, maximum false discovery rate (FDR) on motif similarity ≤0.001, and TF motifs with normalized enrichment score (NES) >3 were considered as the threshold value for the selection of potential associations. TransmiR is a TF–miRNA regulation database, through which regulatory relations between TFs and miRNAs can be identified (Tong et al., 2019). Ultimately, the miRNA–mRNA—-TF regulatory network was constructed utilizing Cytoscape 3.7.1.

## Results

### Identification of DEmRNAs and DE miRNAs (DEmiRs) for TOF

Compared with the normal samples, following quality control and data normalization, a total of 1735 DEmRNAs including 1315 upregulated and 420 downregulated DEmRNAs were identified based on the criteria. miRNA expression analysis showed that a total of seven upregulated miRNAs were identified as significant DEmiRs: miR-155, miR-222, miR-23b, miR-499-3p, miR-1275, miR-93, and miR-187. The volcano plots of the DEmRNAs or DEmiRs are presented in Supplementary Figure S1.

We obtained 6261 targeted genes of the 7 significant DEmiRs from the miRWalk database. Four hundred and fourteen genes were found as the intersection genes between these target genes and the overall upregulated DEmRNAs. Therefore, a total of 529 DEmRNAs, including the 414 upregulated and 115 downregulated genes, were identified as the final sets of significant DEmRNAs (Supplementary Table S1).

### GO and KEGG Pathway Enrichment Analysis of DEmRNAs

**FIGURE 1:**
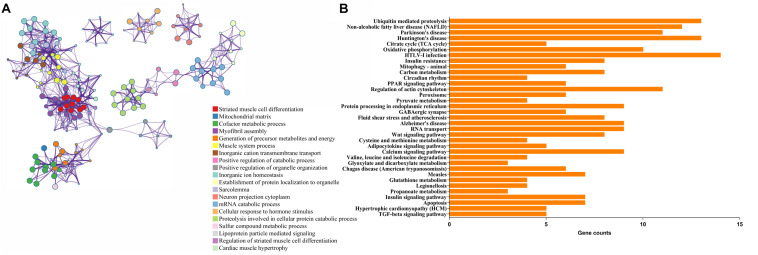
Functional enrichment analyses of differentially expressed genes (DEmRNAs). **(A)** Enriched ontology clusters. Each term is represented by a circle node, where its size is proportional to the number of input genes falling into that term, and its color represents its cluster identity (i.e., nodes of the same color belong to the same cluster). Terms with a similarity score >0.3 are linked by an edge (the thickness of the edge represents the similarity score). **(B)** The top 20 KEGG enrichment analysis for DEmRNAs. The numbers on the *x*-axis are the names of pathways. The numbers on the *y*-axis are gene counts.

Functional and pathway enrichment analyses were applied to explore the pathogenesis in TOF. The DEmRNAs enriched according to the *P*-value are visualized in Figure 1A (Supplementary Table S2). The significantly top five GO terms in DEmRNAs were striated muscle cell differentiation, mitochondrial matrix, cofactor metabolic process, myofibril assembly, and generation of precursor metabolites and energy. The significant top five PPI pathways of DEmRNAs were ubiquitin-mediated proteolysis, non-alcoholic fatty liver disease, Parkinson’s disease, Huntington’s disease, and citrate cycle (Figure 1B and Supplementary Table S3).

### PPI Network Construction and Module Analysis

**FIGURE 2:**
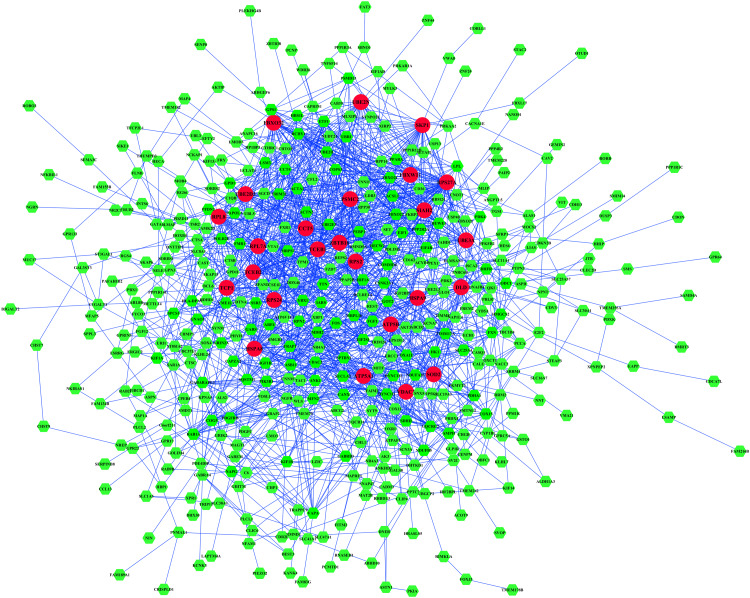
The protein–protein interaction (PPI) network of differentially expressed genes (DEmRNAs). Larger circles with red colors represent hub genes, and smaller hexagons with green colors represent non-hub genes.

**FIGURE 3:**
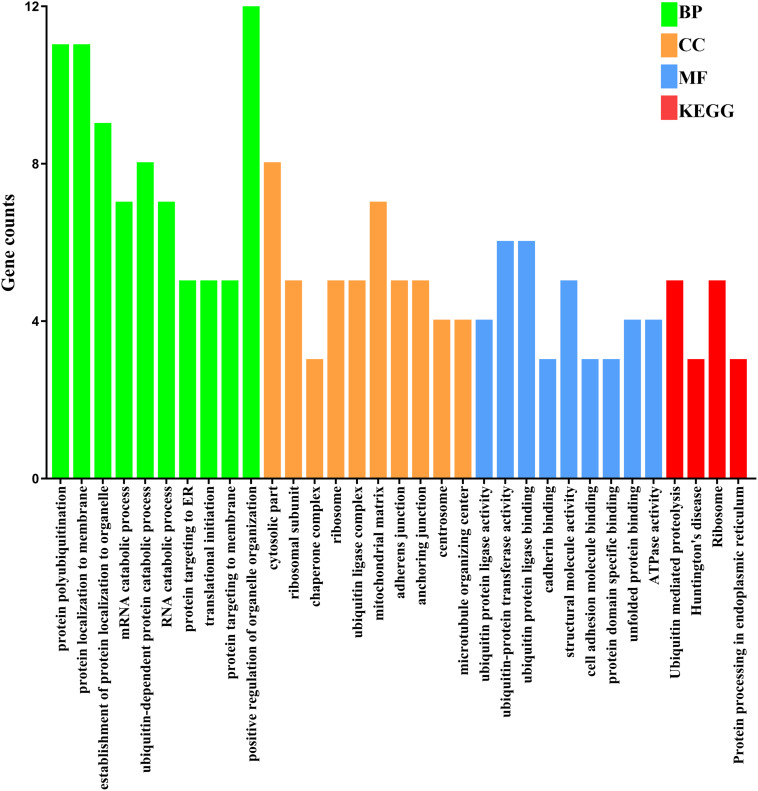
Functional enrichment analyses of the hub genes. The numbers on the *x*-axis are the names of pathways or GO terms. The numbers on the *y*-axis are gene counts. BP, biological progress; CC, cellular component; GO, Gene Ontology; KEGG, Kyoto Encyclopedia of Genes and Genomes; MF, molecular function.

To investigate the relationship of the DEmRNAs, a PPI network was constructed utilizing Cytoscape software based on the STRING database. In total, 448 nodes and 1693 PPI relationships were obtained (Figure 2). Twenty-two genes ranked by top 5% genes with high connectivity were defined as hub genes for TOF, all of which were upregulated (Table 2 and Supplementary Table S4). The 22 hub genes interact directly with 337 edges. GO analysis results showed that hub genes were significantly enriched in ubiquitin–protein transferase activity and ubiquitin protein ligase activity in the molecular function ontology, cytosolic part and ubiquitin ligase complex in cellular components ontology, and protein polyubiquitination and cellular protein catabolic process in biological processes ontology. The results of KEGG analysis showed that hub genes were significantly enriched in ubiquitin-mediated proteolysis and protein processing in endoplasmic reticulum (ER) and ribosomes (Figure 3 and Supplementary Table S5). These hub genes might play a crucial role in TOF and should be studied further.

**TABLE 2:** Hub genes identified in the protein–protein interaction network.

Gene	Degree	Log FC	adj.P.Val	Regulation
*RPS27A*	70	2.2035714	0.0008567	Up
*HSPA8*	46	2.6819216	0.0001913	Up
*SKP1*	38	3.0387064	0.0004551	Up
*HSPA9*	36	3.0767256	0.0001602	Up
*RPS2*	36	2.7716774	0.0008141	Up
*RPL8*	33	2.5430817	0.0005055	Up
*SOD2*	32	3.2653954	7.139E−05	Up
*FBXO32*	31	4.3608082	3.498E−06	Up
*TCP1*	31	3.2699954	0.0001236	Up
*UBE2N*	29	2.2827098	0.0012278	Up
*UBE3A*	28	2.0175226	0.0059344	Up
*PSMC2*	28	2.1476242	1.475E−13	Up
*FBXW11*	27	2.1880644	0.000518	Up
*DLD*	27	4.1483123	4.553E−05	Up
*CCT5*	27	2.5370472	0.0029727	Up
*RPS24*	27	3.2131167	0.000303	Up
*SIAH2*	26	2.4161537	0.0001602	Up
*UBE2D3*	26	2.3856558	0.0007899	Up
*VDAC1*	26	3.971706	4.006E−06	Up
*RPL7A*	26	2.668917	0.00179	Up
*ZBTB16*	25	2.4423304	0.0034434	Up
*UBE2H*	24	2.427039	0.000537	Up
*UBR1*	24	2.065815	0.0006099	Up
*RCHY1*	24	2.8119357	6.861E−05	Up

*adj.P.Val, adjusted *P*-value; log FC, log (Fold Change).*

**FIGURE 4:**
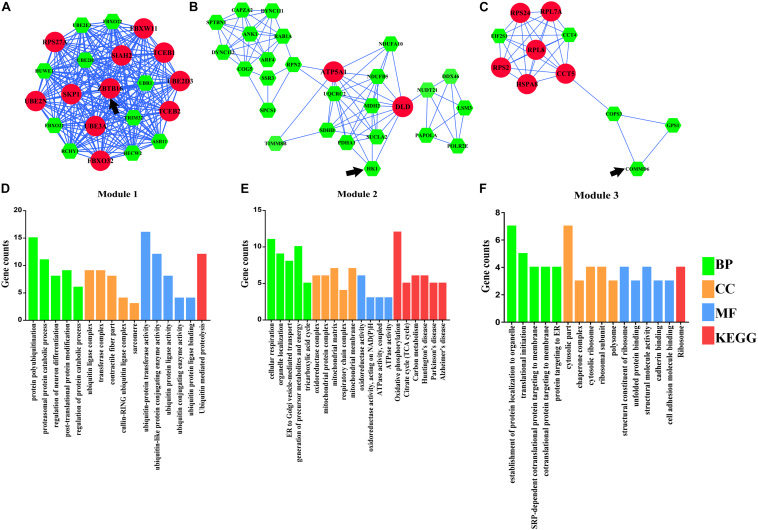
Module analysis of differentially expressed genes (DEmRNAs). **(A)** Module 1. **(B)** Module 2. **(C)** Module 3. **(D)** Functional enrichment analyses of Module 1. **(E)** Functional enrichment analyses of Module 2. **(F)** Functional enrichment analyses of Module 3. Larger circles with red colors represent hub genes, smaller hexagons with green colors represent non-hub genes, and black arrows represent seed genes. BP, biological progress; CC, cellular component; GO, Gene Ontology; KEGG, Kyoto Encyclopedia of Genes and Genomes; MF, molecular function.

A total of three modules from the PPI network satisfied the criteria of an MCODE computed K-score = 4 and degree cut-off = 4 (Figure 4). Fifty-nine MCODE-related genes were identified and which contained the 17 hub genes. Module 1 (Figure 4A) included 21 nodes and 210 PPI pairs; KEGG analysis showed that these genes were significantly enriched in ubiquitin-mediated proteolysis; and GO analysis showed that these genes were mainly associated with protein polyubiquitination, proteasomal protein catabolic process, contractile fiber part, sarcomere, and ubiquitin-protein transferase activity (Figure 4D). Module 2 included 27 nodes and 84 PPI pairs (Figure 4B), and KEGG analysis showed that these genes were enriched in energy metabolism associated pathways (oxidative phosphorylation, citrate cycle, and carbon metabolism), and neurodevelopmental disorders (Parkinson’s disease, Huntington’s disease, and Alzheimer’s disease), and GO terms were primarily in cellular respiration, organelle localization, oxidoreductase complex, mitochondrial protein complex, oxidoreductase activity, and ATPase activity (Figure 4E). Module 3 included 11 nodes and 32 PPI pairs (Figure 4C); KEGG analysis showed that these genes were significantly enriched in ribosomes, and GO analysis revealed that module 3 related genes were mainly associated with establishment of protein localization to organelles, translation initiation, cytosolic part chaperone complex, structural constituents of ribosomes, and unfolded protein binding (Figure 4F). The detailed GO and KEGG pathway analysis information of three modules is shown in Supplementary Table S6.

### miRNA–TF–Target Regulatory Network Analysis

**FIGURE 5:**
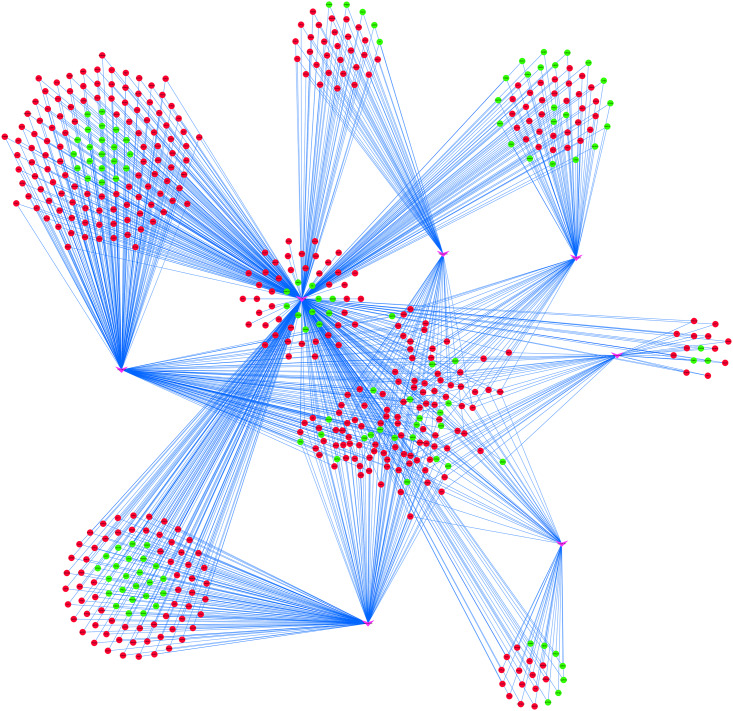
miRNA–target regulatory networks of differentially expressed genes (DEmRNAs). The pink V-shapes represent miRNAs, red circles represent upregulated DEmRNAs, and green circles represent downregulated DEmRNAs.

To further understand the regulatory relationship between DEmiRs and DEmRNAs, the miRNA–target regulation network was constructed as shown in Figure 5. miR-222 had the highest connectivity with 529 target DEmRNAs. Moreover, several miRNAs were predicted to have common targets, for example, miR222 and miR93 have 139 common target genes, and miR222 and miR23b have 90 common target genes (Figure 5).

**FIGURE 6:**
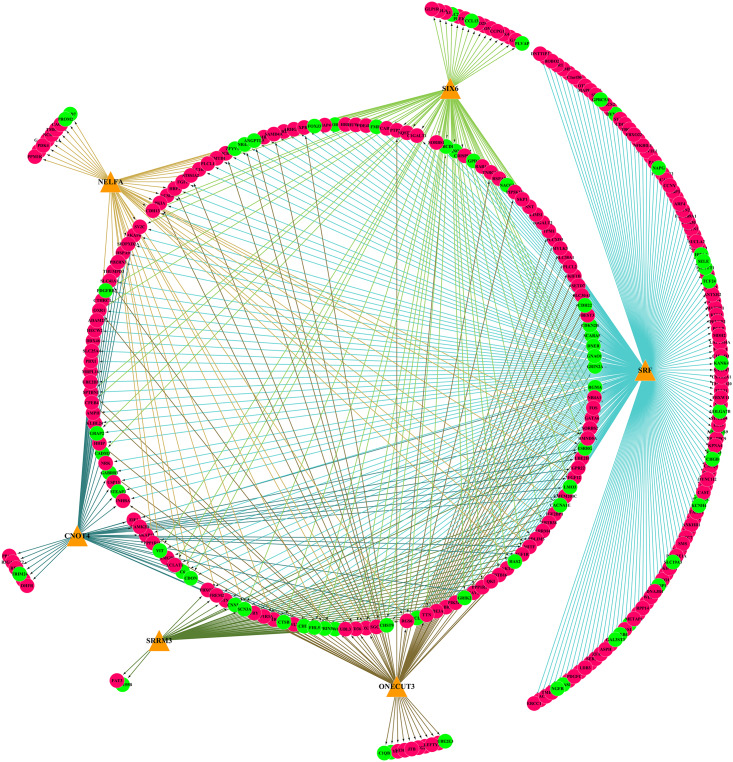
The TF–target regulatory network of differentially expressed genes (DEmRNAs). Orange triangles represent TFs, red circles represent upregulated genes, and green circles represent downregulated genes. TFs, transcription factors.

Six TFs were predicted to regulate DEmRNAs by iRegulon analysis, including SRF, CNOT4, SIX6, SRRM3, NELFA, and ONECUT3. To determine the regulatory connections between TFs and DEmRNAs, the TF–target regulation network of 302 nodes and 556 interaction pairs was constructed (Figure 6), SRF targeted 254 genes, CNOT4 targeted 60 genes, SIX6 targeted 84 genes, SRRM3 targeted 44 genes, NELFA targeted 46 genes, ONECUT3 targeted 68 genes. The top 20 nodes with the highest degree are listed in Table 3, including HSPA8, SKP1, HSPA9, FBXO32, UBE3A, FBXW11, VDAC1, UBE2D3, SIAH2, ZBTB16, UBE2H, HIF1A, UQCRC2, MDH2, SQSTM1, FBXO22, ASB11, HUWE1, HECW2, and RAB1A, which were coregulated by the three TFs.

**TABLE 3:** Top 20 nodes with higher degree in transcription factor–target regulatory network.

**Node name**	**Regulation**	**Degree**	**TF**
HSPA8	Up	46	SIX6
SKP1	Up	38	SIX6
HSPA9	Up	36	SRF
FBXO32	Up	31	SRF
UBE3A	Up	28	SRF
FBXW11	Up	27	SRF
VDAC1	Up	26	SRF
UBE2D3	Up	26	SRF
SIAH2	Up	26	SRF
ZBTB16	Up	25	SIX6
UBE2H	Up	24	SRF
HIF1A	Down	23	SRF
UQCRC2	Up	23	SIX6
MDH2	Up	23	SRF
SQSTM1	Up	23	SIX6
FBXO22	Up	23	SRF
ASB11	Up	23	NELFA
HUWE1	Up	22	SIX6
HECW2	Up	22	SRF
RAB1A	Up	21	SIX6

*TF, transcription factor.*

**FIGURE 7:**
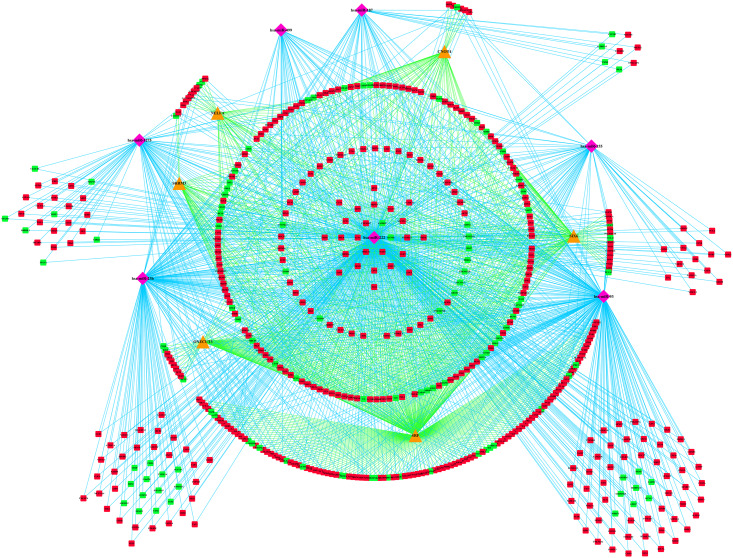
miRNA–mRNA–TF regulatory network of differentially expressed genes (DEmRNAs). The pink diamonds represent miRNAs, orange triangles represent TFs, red circles represent upregulated genes, and green circles represent downregulated genes. TFs, transcription factors.

TransmiR analysis revealed that SRF regulated miR-23b and miR-93; miR222 and miR-23b are regulated by MITF, but also target MITF; BCL6 is the target of miR-93 and miR-155, which also regulates miR-93 and miR-155; ZBTB16 could repress miR-222, while FOSL1 could activate miR-222. Finally, the miRNA–mRNA–TF regulatory network was established with 7 miRNAs, 529 DEmRNAs, and 6 TFs through Cytoscape 3.7.1 (Figure 7).

## Discussion

Heart development is an extremely complex process involving networks of various signaling pathways, the downstream TFs, protein-coding genes, and posttranscriptional regulation for cardiac myogenesis, morphogenesis, and contractility. Owing to the lack of specific molecule mechanisms, genetic counseling and prenatal diagnosis of TOF have been a major challenge. Therefore, it is imperative to explore the specific molecular mechanism for TOF, which will be valuable for risk assessment and even prognosis prediction. Recently, certain dysregulated mRNAs, miRNAs, and TFs have been reported in TOF individually. Notably, transcriptional and posttranscriptional regulation plays important roles in TOF development.

In our study, an integrative analysis of microarray and RNA-sequencing datasets was conducted to identify the regulatory network underlying TOF. A total of 529 DEmRNAs, 7 DEmiRs, and 6 TFs were identified as significant elements for the TOF. Based on 529 DEmRNAs, we obtained 22 hub genes and three significant modules from PPI networks using MCODE analysis. Eleven hub genes were significantly enriched in protein polyubiquitination, including *SKP1*, *FBXO32*, *UBE3A*, *PSMC2*, *RPS27A*, *SIAH2*, *UBE2D3*, *UBE2N*, *FBXW11*, *DLD*, and *ZBTB16*. The ubiquitin–proteasome system (UPS) is important for the regulation of cellular protein degradation and synthesis (Al-Hassnan et al., 2016). Impaired UPS has been involved in the etiology of human cardiovascular diseases, such as cardiomyopathy (CMP), heart failure, and cardiac ischemia. Skp1/Cul1/F-box (SCF) ubiquitin E3 ligase complex plays a crucial role in ubiquitination of cardiac proteins (Jang et al., 2011). *FBXO32*, which encodes a muscle-specific ubiquitin ligase, has been implicated in the pathogenesis of CMP through the UPS (Al-Hassnan et al., 2016). *UBE3A* is one of the important members of UPS; *Ube3a* mutant mice had deficits in Ca^2+^/calmodulin-dependent kinase II (CaMKII), while CaMKII played a vital role in cardiac hypertrophy (Cheng et al., 2019). *ZBTB16*, a Kruppel-like zinc finger protein, highly expressed in the heart, is an important TF that regulates cardiac hypertrophy through the Ang II receptor 2 in response to angiotensin II (Wang et al., 2012). GO function annotation also indicates that many hub genes (*HSPA8*, *PSMC2*, *RPL7A*, *RPL8*, *RPS2*, *RPS24*, *RPS27A*, *HSPA9*, *TCP1*, *CCT5*) are significantly enriched in regulation of transcription, posttranscriptional regulation and translational regulation, including mRNA catabolic process, RNA catabolic process, ribosomes, translational initiation, nuclear-transcribed mRNA catabolic process, regulation of mRNA stability, DNA biosynthetic process, and rRNA processing. Elevated rates of protein synthesis have been demonstrated to contribute to cardiomyocyte hypertrophy. Accelerated rates of protein synthesis can be achieved by altering the translational efficiency of existing ribosomes and/or by accelerating the rate of synthesis of new ribosomes, indicating a crucial role for ribosome biogenesis in the regulation of cardiac growth (Brandenburger et al., 2003). Abnormal expression of ribosomes can affect the development and function of the cardiomyocyte and may lead to right ventricular hypertrophy of TOF. Members of the heat shock protein 70 family (*HSPA8* and *HSPA9*) have also been proven to be essential to maintain cardiomyocyte protein homeostasis (Fang et al., 2017). Underexpression of *HSPA8* and *HSPA9* will disrupt cardiomyocyte homeostasis and interfere with critical cellular functions by small aggregates of proteins, which finally contributes to heart failure and hereditary heart diseases. However, the biochemical property and the molecular function of other hub genes have not been fully elucidated, and further functional studies are crucial to better understand the mechanism underlying the pathogenesis of TOF.

Gene Ontology analysis of DEmRNAs showed that DEmRNAs are mainly involved in striated muscle cell differentiation, mitochondrial matrix, cofactor metabolic process, myofibril assembly, and generation of precursor metabolites and energy, and these biological processes mainly contribute to cardiac cell division and differentiation. KEGG pathway analysis demonstrated abnormal molecular expression of several pathways may contribute to the pathogenesis of TOF, such as ubiquitin-mediated proteolysis pathways, energy metabolism associated pathways (citrate cycle, cysteine and methionine metabolism, glycolysis/gluconeogenesis, and PPAR signaling pathway), and pathways associated with neurodevelopmental disorders (Parkinson’s disease, Huntington’s disease, and Alzheimer’s disease). Ubiquitin-mediated proteolysis is essential to maintain the integrity of cellular proteins that make up sarcomeres, mitochondria, ER, and membranes and therefore is critical to cardiomyocyte survival and functioning (Pagan et al., 2013; Li et al., 2018). Insufficient ubiquitin-mediated proteolysis might be a pathogenic factor leading to cardiac pathogenesis (Li et al., 2018). Myocardial energy metabolism is an important determinant of cardiac structure and function (Finck, 2007). Energy metabolism associated pathways affect the normal cellular biological processes, including cell survival/death, differentiation/proliferation, and DNA repair (Dhar-Chowdhury et al., 2007; Hanahan and Weinberg, 2011; Tran and Wang, 2019). Pathological alterations of energy metabolism pathways could result in impaired signaling transduction, perturbed ion and redox homeostasis, and contractile dysfunction. Future studies in the specific role of energy metabolism will shed new light on the pathogenesis of TOF. Fifteen genes, including *SLC25A4*, *ATP5F1A*, *ATP5F1B*, *NDUFA10*, *NDUFB5*, *POLR2E*, *REST*, *SDHD*, *SOD2*, *UQCRC2*, *LPL*, *VDAC1*, *VDAC2*, *UQCR11*, and *GRIN2A*, were also enriched in neurodevelopmental disorders associated pathways. New research shows neurodevelopmental disorders occur in 10% of all children with CHD and in 50% of children with severe CHD (Marino et al., 2012), and *de novo* mutations account for at least 20% of CHD patients with associated extracardiac or neurodevelopmental abnormalities (Homsy et al., 2015), indicating a striking shared genetic etiology between TOF and neurodevelopmental disorders.

Among the six TFs predicted in the present study, SRF has been previously reported to contribute to differentiation, maturation, and homeostasis of cardiomyocytes, Srf knockout mice show severe defects vascular smooth muscle cells lacking organizing actin/intermediate filament bundles and die at the gastrulation stage (Schlesinger et al., 2011). CNOT4 functions as a ubiquitin-protein ligase (E3) (Deshaies and Joazeiro, 2009), interacting with a subset of ubiquitin−conjugating enzymes (E2s). SIX6 can mediate the repression of p27/Kip1, an inhibitor of the cell cycle G1/S transition, thereby regulating cellular organ−specific proliferation (Conte et al., 2005). SRRM3 is a alternative splicing regulator required for motor coordination (Nakano et al., 2019), potentially related to myocardial systolic motion. NELFA, a member of the NELF, cause promoter proximal pausing on the HSP70 gene (Wu et al., 2003), while *HSPA8* and *HSPA9* are identified hub genes of higher degrees. ONECUT3 is essential in multiple tissues for proper specification and development of unique cell types (Kropp and Gannon, 2016). However, whether these TFs are implicated in the pathogenesis of TOF can be investigated in future studies.

MicroRNAs, one of the most important endogenous epigenetic factors, inhibit posttranscriptional gene expression of target genes. Recently, several studies have revealed miRNAs as governing gene expression during cardiac development (Small and Olson, 2011). Seven upregulated DEmiRs were identified in the present study, including miR-499, miR-155, miR-23b, miR-222, miR-1275, miR-93, and miR-187. Among of them, miR-499, miR-155, miR-23b, and miR-222 have been demonstrated as participating in cardiac development and function. miR-499 regulates the mitochondrial fission machinery in cardiomyocytes by suppressing calcineurin-mediated dephosphorylation of DRP1 (Wang et al., 2011). Similarly, miR-1275 was found to correlate with decreased mitochondrial membrane potential (Nymark et al., 2015). miR-155 is a critical microRNA that represses MEF2A expression (Seok et al., 2011), and *Mef2a* knockout mice exhibit dilated right ventricle, myofibrillar fragmentation, mitochondrial disorganization, and cardiac sudden death (Naya et al., 2002). miR-222 is upregulated in ventricular outflow tract tissues from infants with non-syndromic TOF, and miR-222 is involved in proliferation and differentiation of cardiomyocytes in *in vitro* experiments (Zhang et al., 2013). miR-23b was found upregulated in cyanotic CHD patients, and the upregulated miR-23b increases cardiomyocyte apoptosis and reduces cell growth under hypoxic conditions (Li et al., 2017). miR-93 was identified as highly expressed in the fetal mouse heart at four key time points (embryonic day E12.5, E14.5, E16.5, and E18.5) (Cao et al., 2012). miR-187 and its target genes *Itpkc*, *Tbl1xr1*, and *Lrrc59* were reported to play regulatory roles in cardiomyocyte apoptosis and cardiac inflammation (Ektesabi, 2019). Therefore, we speculate that these miRNAs mainly contribute to abnormal cardiac cell division, differentiation, and apoptosis for TOF pathogenesis.

## Conclusion

In summary, we performed an integrated analysis, based on four microarray datasets and one RNA-seq dataset, to identify specific TFs, miRNAs, and mRNAs that may play a pivotal role in TOF. Moreover, we established a TF–miRNA–mRNA network in TOF. However, there are certain limitations in our present study. The sample number of TOF specimens from patients was relatively small, which may reduce the credibility of the miRNA–mRNA–TF coregulatory network analysis. In addition, the present study lacked further experiments to verify the analysis results as a solid foundation. All in all, the current research provides a comprehensive perspective of regulatory mechanism networks underlying TOF and also identifies potential molecule targets of genetic counseling and prenatal diagnosis.

## Data Availability Statement

The datasets generated for this study can be found in the Gene Expression Omnibus (GEO): GSE35776, GSE36761, GSE26125, GSE40128, GSE35490.

## Author Contributions

FL conceived and designed the experiments. GY, BZ, BW, and QF analyzed and interpreted the data. GY and BZ wrote the manuscript. All the authors reviewed the manuscript.

## Conflict of Interest

The authors declare that the research was conducted in the absence of any commercial or financial relationships that could be construed as a potential conflict of interest.

## Supplementary Material

FIGURE S1 Volcano plots of differentially expressed genes or microRNAs. The *x*-axis represents the fold change (log-scaled), and the *y*-axis represents the adjusted *P*-value (log-scaled). Each symbol represents a different gene. The red color of the symbols means upregulated, whereas the green color of the symbols means downregulated. **(A)** Volcano plots of GSE26125. **(B)** Volcano plots of GSE35776. **(C)** Volcano plots of GSE36761. **(D)** Volcano plots of GSE40128. **(E)** Volcano plots of GSE35490.

TABLE S1 Significant differentially expressed genes.

TABLE S2 GO enrichment analysis of differentially expressed genes.

TABLE S3 KEGG pathway analysis of differentially expressed genes.

TABLE S4 Detailed information on Hub genes.

TABLE S5 GO and KEGG enrichment analysis of hub genes.

TABLE S6 GO and KEGG enrichment analysis of modules 1, 2, and 3.

## References

[B1] Al-Hassnan Z. N. Shinwari Z. M. Wakil S. M. Tulbah S. Mohammed S. Rahbeeni Z. (2016). A substitution mutation in cardiac ubiquitin ligase, FBXO32, is associated with an autosomal recessive form of dilated cardiomyopathy. *BMC Med. Genet.* 17:3. 10.1186/s12881-016-0267-5 26768247 PMC4714499

[B2] Ashburner M. Ball C. A. Blake J. A. Botstein D. Butler H. Cherry J. M. (2000). Gene ontology: tool for the unification of biology. The Gene Ontology Consortium. *Nat. Genet.* 25 25–29. 10.1038/75556 10802651 PMC3037419

[B3] Bader G. D. Hogue C. W. (2003). An automated method for finding molecular complexes in large protein interaction networks. *BMC Bioinformatics* 4:2. 10.1186/1471-2105-4-2 12525261 PMC149346

[B4] Bailliard F. Anderson R. H. (2009). Tetralogy of Fallot. *Orphanet J. Rare Dis.* 4:2. 10.1186/1750-1172-4-2 PMC2651859 19144126

[B5] Bittel D. C. Butler M. G. Kibiryeva N. Marshall J. A. Chen J. Lofland G. K. (2011). Gene expression in cardiac tissues from infants with idiopathic conotruncal defects. *BMC Med. Genomics* 4:1. 10.1186/1755-8794-4-1 21208432 PMC3023653

[B6] Bittel D. C. Kibiryeva N. Marshall J. A. O’Brien J. E. (2014). MicroRNA-421 dysregulation is associated with tetralogy of fallot. *Cells* 3 713–723. 10.3390/cells3030713 25257024 PMC4197626

[B7] Bolger A. M. Lohse M. Usadel B. (2014). Trimmomatic: a flexible trimmer for Illumina sequence data. *Bioinformatics* 30 2114–2120. 10.1093/bioinformatics/btu170 24695404 PMC4103590

[B8] Brandenburger Y. Arthur J. F. Woodcock E. A. Du X. J. Gao X. M. Autelitano D. J. (2003). Cardiac hypertrophy in vivo is associated with increased expression of the ribosomal gene transcription factor UBF. *FEBS Lett.* 548 79–84. 10.1016/s0014-5793(03)00744-0 12885411

[B9] Cao L. Kong L. P. Yu Z. B. Han S. P. Bai Y. F. Zhu J. (2012). microRNA expression profiling of the developing mouse heart. *Int. J. Mol. Med.* 30 1095–1104. 10.3892/ijmm.2012.1092 22895573

[B10] Carvalho B. S. Irizarry R. A. (2010). A framework for oligonucleotide microarray preprocessing. *Bioinformatics* 26 2363–2367. 10.1093/bioinformatics/btq431 20688976 PMC2944196

[B11] Cheng K. C. Li Y. Chang W. T. Chen Z. C. Cheng J. T. Tsai C. C. (2019). Ubiquitin-protein ligase E3a (UBE3A) as a new biomarker of cardiac hypertrophy in cell models. *J. Food Drug Anal.* 27 355–364. 10.1016/j.jfda.2018.08.002 30648591 PMC9298619

[B12] Clough E. Barrett T. (2016). The gene expression omnibus database. *Methods Mol. Biol.* 1418 93–110. 27008011 10.1007/978-1-4939-3578-9_5 PMC4944384

[B13] Conte I. Morcillo J. Bovolenta P. (2005). Comparative analysis of Six 3 and Six 6 distribution in the developing and adult mouse brain. *Dev. Dyn.* 234 718–725. 10.1002/dvdy.20463 15973738

[B14] Deshaies R. J. Joazeiro C. A. (2009). RING domain E3 ubiquitin ligases. *Annu. Rev. Biochem.* 78 399–434. 10.1093/hmg/ddw362 19489725

[B15] Dhar-Chowdhury P. Malester B. Rajacic P. Coetzee W. A. (2007). The regulation of ion channels and transporters by glycolytically derived ATP. *Cell Mol. Life. Sci* 64 3069–3083. 10.1007/s00018-007-7332-3 17882378 PMC11135988

[B16] Diboun I. Wernisch L. Orengo C. A. Koltzenburg M. (2006). Microarray analysis after RNA amplification can detect pronounced differences in gene expression using limma. *BMC Genomics* 7:252. 10.1186/1471-2164-7-252 17029630 PMC1618401

[B17] Dobin A. Davis C. A. Schlesinger F. Drenkow J. Zaleski C. Jha S. (2013). STAR: ultrafast universal RNA-seq aligner. *Bioinformatics* 29 15–21. 10.1093/bioinformatics/bts635 23104886 PMC3530905

[B18] Dweep H. Gretz N. (2015). miRWalk2.0: a comprehensive atlas of microRNA-target interactions. *Nat. Methods* 12:697. 10.1038/nmeth.3485 26226356

[B19] Ektesabi A. M. (2019). Regulation of mir-187b in endotoxemic primary cardiomyocytes, and septic murine hearts treated with mesenchymal stromal/stem cells. *Can. J. Cardiol.* 35:S48.

[B20] Fang X. Bogomolovas J. Wu T. Zhang W. Liu C. Veevers J. (2017). Loss-of-function mutations in co-chaperone BAG3 destabilize small HSPs and cause cardiomyopathy. *J. Clin. Invest.* 127 3189–3200. 10.1172/JCI94310 28737513 PMC5531406

[B21] Finck B. N. (2007). The PPAR regulatory system in cardiac physiology and disease. *Cardiovasc. Res.* 73 269–277. 10.1016/j.cardiores.2006.08.023 17010956

[B22] Gautier L. Cope L. Bolstad B. M. Irizarry R. A. (2004). affy–analysis of Affymetrix GeneChip data at the probe level. *Bioinformatics* 20 307–315. 10.1093/bioinformatics/btg405 14960456

[B23] Grunert M. Dorn C. Schueler M. Dunkel I. Schlesinger J. Mebus S. (2014). Rare and private variations in neural crest, apoptosis and sarcomere genes define the polygenic background of isolated Tetralogy of Fallot. *Hum. Mol. Genet.* 23 3115–3128. 10.1093/hmg/ddu021 24459294

[B24] Hanahan D. Weinberg R. A. (2011). Hallmarks of cancer: the next generation. *Cell* 144 646–674. 21376230 10.1016/j.cell.2011.02.013

[B25] Homsy J. Zaidi S. Shen Y. Ware J. S. Samocha K. E. Karczewski K. J. (2015). De novo mutations in congenital heart disease with neurodevelopmental and other congenital anomalies. *Science* 350 1262–1266. 10.1126/science.aac9396 26785492 PMC4890146

[B26] Jang J. W. Lee W. Y. Lee J. H. Moon S. H. Kim C. H. Chung H. M. (2011). A novel Fbxo25 acts as an E3 ligase for destructing cardiac specific transcription factors. *Biochem. Biophys. Res. Commun.* 410 183–188. 10.1016/j.bbrc.2011.05.011 21596019

[B27] Janky R. Verfaillie A. Imrichova H. Van de Sande B. Standaert L. Christiaens V. (2014). iRegulon: from a gene list to a gene regulatory network using large motif and track collections. *PLoS Comput. Biol.* 10:e1003731. 10.1371/journal.pcbi.1003731 25058159 PMC4109854

[B28] Kanehisa M. Goto S. (2000). KEGG: kyoto encyclopedia of genes and genomes. *Nucleic Acids Res.* 28 27–30. 10.1038/gene.2015.7 10592173 PMC102409

[B29] Kropp P. A. Gannon M. (2016). Onecut transcription factors in development and disease. *Trends Dev. Biol.* 9 43–57. 28018056 PMC5176019

[B30] Lalani S. R. Belmont J. W. (2014). Genetic basis of congenital cardiovascular malformations. *Eur. J. Med. Genet.* 57 402–413. 10.1016/j.ejmg.2014.04.010 24793338 PMC4152939

[B31] Li H. Luo M. Zheng J. Luo J. Zeng R. Feng N. (2017). An artificial neural network prediction model of congenital heart disease based on risk factors: A hospital-based case. (-)control study. *Medicine* 96:e6090. 10.1097/MD.0000000000006090 28178169 PMC5313026

[B32] Li J. Johnson J. A. Su H. (2018). Ubiquitin and Ubiquitin-like proteins in cardiac disease and protection. *Curr. Drug Targets* 19 989–1002. 10.2174/1389450117666151209114608 26648080 PMC4899309

[B33] Liao Y. Smyth G. K. Shi W. (2014). featureCounts: an efficient general purpose program for assigning sequence reads to genomic features. *Bioinformatics* 30 923–930. 10.1093/bioinformatics/btt656 24227677

[B34] Liu N. Bezprozvannaya S. Williams A. H. Qi X. Richardson J. A. Bassel-Duby R. (2008). microRNA-133a regulates cardiomyocyte proliferation and suppresses smooth muscle gene expression in the heart. *Genes Dev.* 22 3242–3254. 10.1101/gad.1738708 19015276 PMC2600761

[B35] Liu N. Olson E. N. (2010). MicroRNA regulatory networks in cardiovascular development. *Dev. Cell* 18 510–525. 10.1016/j.devcel.2010.03.010 20412767 PMC2922691

[B36] Love M. I. Huber W. Anders S. (2014). Moderated estimation of fold change and dispersion for RNA-seq data with DESeq2. *Genome Biol.* 15:550. 10.1186/s13059-014-0550-8 25516281 PMC4302049

[B37] Marino B. S. Lipkin P. H. Newburger J. W. Peacock G. Gerdes M. Gaynor J. W. (2012). Neurodevelopmental outcomes in children with congenital heart disease: evaluation and management: a scientific statement from the American Heart Association. *Circulation* 126 1143–1172. 10.1161/CIR.0b013e318265ee8a 22851541

[B38] Nakano Y. Wiechert S. Banfi B. (2019). Overlapping activities of two neuronal splicing factors switch the GABA effect from excitatory to inhibitory by regulating REST. *Cell Rep* 27:860-871.e8. 10.1016/j.celrep.2019.03.072 30995482 PMC6556397

[B39] Naya F. J. Black B. L. Wu H. Bassel-Duby R. Richardson J. A. Hill J. A. (2002). Mitochondrial deficiency, and cardiac sudden death in mice lacking the MEF2A transcription factor. *Nat. Med.* 8 1303–1309. 10.1038/nm789 12379849

[B40] Nymark P. Wijshoff P. Cavill R. van Herwijnen M. Coonen M. L. Claessen S. (2015). Extensive temporal transcriptome and microRNA analyses identify molecular mechanisms underlying mitochondrial dysfunction induced by multi-walled carbon nanotubes in human lung cells. *Nanotoxicology* 9 624–635. 10.3109/17435390.2015.1017022 25831214

[B41] O’Brien J. E. Jr. Kibiryeva N. Zhou X. G. Marshall J. A. Lofland G. K. Artman M. (2012). Noncoding RNA expression in myocardium from infants with tetralogy of Fallot. *Circ. Cardiovasc. Genet.* 5 279–286. 10.1161/CIRCGENETICS.111.961474 22528145

[B42] Olson E. N. (2006). Gene regulatory networks in the evolution and development of the heart. *Science* 313 1922–1927. 10.1101/gad.1485706 17008524 PMC4459601

[B43] Pagan J. Seto T. Pagano M. Cittadini A. (2013). Role of the ubiquitin proteasome system in the heart. *Circ. Res.* 112 1046–1058. 10.1161/CIRCRESAHA.112.300521 23538275

[B44] Scardoni G. Petterlini M. Laudanna C. (2009). Analyzing biological network parameters with CentiScaPe. *Bioinformatics* 25 2857–2859. 10.1093/bioinformatics/btp517 19729372 PMC2781755

[B45] Schlesinger J. Schueler M. Grunert M. Fischer J. J. Zhang Q. Krueger T. (2011). The cardiac transcription network modulated by Gata4, Mef2a, Nkx2.5, Srf, histone modifications, and microRNAs. *PLoS Genet.* 7:e1001313. 10.1371/journal.pgen.1001313 21379568 PMC3040678

[B46] Seok H. Y. Tatsuguchi M. Callis T. E. He A. Pu W. T. Wang D. Z. (2011). miR-155 inhibits expression of the MEF2A protein to repress skeletal muscle differentiation. *J. Biol. Chem.* 286 35339–35346. 10.1074/jbc.M111.273276 21868385 PMC3195620

[B47] Shannon P. Markiel A. Ozier O. Baliga N. S. Wang J. T. Ramage D. (2003). Cytoscape: a software environment for integrated models of biomolecular interaction networks. *Genome Res.* 13 2498–2504. 10.1101/gr.1239303 14597658 PMC403769

[B48] Slezak-Prochazka I. Durmus S. Kroesen B. J. van den Berg A. (2010). MicroRNAs, macrocontrol: regulation of miRNA processing. *RNA* 16 1087–1095. 10.1261/rna.1804410 20423980 PMC2874160

[B49] Small E. M. Olson E. N. (2011). Pervasive roles of microRNAs in cardiovascular biology. *Nature* 469 336–342. 10.1038/nature09783 21248840 PMC3073349

[B50] Szklarczyk D. Franceschini A. Wyder S. Forslund K. Heller D. Huerta-Cepas J. (2015). STRING v10: protein-protein interaction networks, integrated over the tree of life. *Nucleic Acids Res.* 43 D447–D452. 10.1093/nar/gku1003 25352553 PMC4383874

[B51] Tong Z. Cui Q. Wang J. Zhou Y. (2019). TransmiR v2.0: an updated transcription factor-microRNA regulation database. *Nucleic Acids Res.* 47 D253–D258. 10.1093/nar/gky1023 30371815 PMC6323981

[B52] Tran D. H. Wang Z. V. (2019). Glucose metabolism in cardiac hypertrophy and heart failure. *J. Am. Heart Assoc.* 8 e012673. 10.1161/JAHA.119.012673 31185774 PMC6645632

[B53] Ventura A. Young A. G. Winslow M. M. Lintault L. Meissner A. Erkeland S. J. (2008). Targeted deletion reveals essential and overlapping functions of the miR-17 through 92 family of miRNA clusters. *Cell* 132 875–886. 10.1016/j.cell.2008.02.019 18329372 PMC2323338

[B54] Wang B. Shi G. Zhu Z. Chen H. Fu Q. (2018). Sexual difference of small RNA expression in Tetralogy of Fallot. *Sci. Rep.* 8:12847. 10.1038/s41598-018-31243-6 30150777 PMC6110777

[B55] Wang J. X. Jiao J. Q. Li Q. Long B. Wang K. Liu J. P. (2011). miR-499 regulates mitochondrial dynamics by targeting calcineurin and dynamin-related protein-1. *Nat. Med.* 17 71–78. 10.1038/nm.2282 21186368

[B56] Wang N. Frank G. D. Ding R. Tan Z. Rachakonda A. Pandolfi P. P. (2012). Promyelocytic leukemia zinc finger protein activates GATA4 transcription and mediates cardiac hypertrophic signaling from angiotensin II receptor 2. *PLoS One* 7:e35632. 10.1371/journal.pone.0035632 22558183 PMC3338737

[B57] Wu C. H. Yamaguchi Y. Benjamin L. R. Horvat-Gordon M. Washinsky J. Enerly E. (2003). NELF and DSIF cause promoter proximal pausing on the hsp70 promoter in Drosophila. *Genes Dev.* 17 1402–1414. 10.1101/gad.1091403 12782658 PMC196072

[B58] Zaret K. S. Carroll J. S. (2011). Pioneer transcription factors: establishing competence for gene expression. *Genes Dev.* 25 2227–2241. 10.1101/gad.176826.111 22056668 PMC3219227

[B59] Zhang J. Chang J. J. Xu F. Ma X. J. Wu Y. Li W. C. (2013). MicroRNA deregulation in right ventricular outflow tract myocardium in nonsyndromic tetralogy of fallot. *Can. J. Cardiol.* 29 1695–1703. 10.1016/j.cjca.2013.07.002 24140236

[B60] Zhou Y. Zhou B. Pache L. Chang M. Khodabakhshi A. H. Tanaseichuk O. (2019). Metascape provides a biologist-oriented resource for the analysis of systems-level datasets. *Nat. Commun.* 10:1523. 10.1038/s41467-019-09234-6 30944313 PMC6447622

