# Supplementary figures and images for: Identification of miRNA–mRNA–TFs Regulatory Network and Crucial Pathways Involved in Tetralogy of Fallot
Source: Front Genet. 2020 Jun 12;11:552. doi: 10.3389/fgene.2020.00552 (PMC7303929; doi:10.3389/fgene.2020.00552)

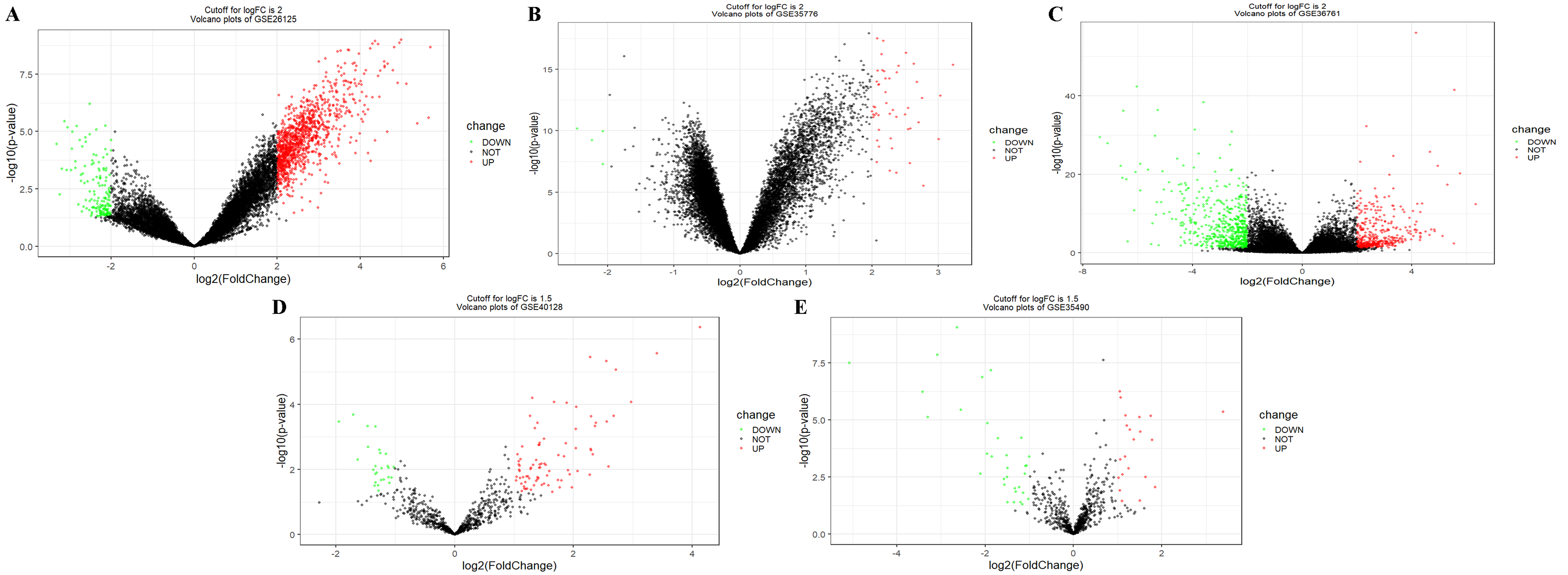

Supplement: FIGURE S1 — Volcano plots of differentially expressed genes or microRNAs. The x-axis represents the fold change (log-scaled), and the y-axis represents the adjusted P-value (log-scaled). Each symbol represents a different gene. The red color of the symbols means upregulated, whereas the green color of the symbols means downregulated. (A) Volcano plots of GSE26125. (B) Volcano plots of GSE35776. (C) Volcano plots of GSE36761. (D) Volcano plots of GSE40128. (E) Volcano plots of GSE35490. [file Image_1.TIF]
